# Vitamin D3 Attenuates Viral-Induced Inflammation and Fibrotic Responses in Bronchial Smooth Muscle Cells

**DOI:** 10.3389/fimmu.2021.715848

**Published:** 2021-08-26

**Authors:** Maria Plesa, Mellissa Gaudet, Andrea Mogas, Nour Jalaleddine, Andrew Halayko, Saba Al Heialy, Qutayba Hamid

**Affiliations:** ^1^Translational Research in Respiratory Diseases, Meakins-Christie Laboratories, Research Institute of the McGill University Health Center, Montréal, QC, Canada; ^2^Mohammed Bin Rashid University of Medicine and Health Sciences, College of Medicine, Dubai, United Arab Emirates; ^3^Departments of Physiology and Pathophysiology, Children’s Hospital Research Institute of Manitoba, Winnipeg, MB, Canada; ^4^Faculty of Medicine, McGill University, Montréal, QC, Canada; ^5^College of Medicine, University of Sharjah, Sharjah, United Arab Emirates

**Keywords:** asthma, COPD, vitamin D3, polyI:C, pulmonary fibrosis

## Abstract

Toll-like receptor 3 (TLR3) activation by viral infections plays a key role in promoting inflammatory immune responses that contribute to pulmonary fibrosis in chronic inflammatory respiratory diseases. Vitamin D3 has been shown to be beneficial to patients with asthma and chronic obstructive pulmonary disease (COPD) through its anti-inflammatory and anti-fibrotic properties. Smooth muscle cells are one of the major contributors to airway remodeling in asthma and COPD. We therefore aimed to investigate the effect of vitamin D3 treatment on viral-induced TLR3 responses in Bronchial Smooth Muscle Cells (BSMCs) as a mechanism contributing to pulmonary fibrosis in asthma and COPD. Primary BSMCs from patients with asthma (n=4), COPD (n=4), and healthy control subjects (n=6) were treated with polyinosinic: polycytidylic acid (polyI:C), TLR3 agonist in the presence or absence of vitamin D3 (1,25D3). Here we report the mRNA expression and protein levels of pro-inflammatory and pro-fibrotic markers (IL-6, IFN-β1, CCL2/MCP-1, fibronectin 1 and type I collagen) among BSMCs groups: asthma, COPD, and healthy controls. We show that at the baseline, prior to polyI:C stimulation, asthma and COPD BSMCs presented increased pro-inflammatory and pro-fibrotic state compared to healthy control subjects, as measured by quantitative PCR and immunoassays (ELISA/Flow Cytometry. Ligation of TLR3 by polyI:C in BSMCs was associated with increased *TLR3* mRNA expression, and 1,25D3 treatment significantly reduced its expression. In addition, 1,25D3 decreased the expression of *IL-6*, *IFN-β1*, *CCL2*, *FN1* and *COL1A1* induced by polyI:C in BSMCs. The regulatory effect of 1,25D3 treatment on polyI:C-stimulated BSMCs was further confirmed at protein levels. Our findings suggest that vitamin D3 attenuates TLR3 agonist-induced inflammatory and fibrotic responses in BSMCs and support the clinical relevance of vitamin D3 supplementation in patients with viral infections having chronic respiratory diseases, such as asthma and COPD.

## Introduction

Pulmonary fibrosis is a complex process that involves activation of several pattern recognition receptors (PRRs) and an interplay of many cell types that contribute directly or indirectly to airway remodeling. Increased thickness of the airway smooth muscle (ASM) layer due to hypertrophy and hyperplasia of ASM cells is a feature of airway remodeling in both asthma and chronic obstructive pulmonary disease (COPD) (1, 2). Bronchial smooth muscle cells (BSMCs) have been described as an important source of many pro-inflammatory and pro-fibrotic mediators (3). Therefore, increased proliferation of smooth muscle and excessive secretion may contribute to increased airflow obstruction and extracellular matrix (ECM) deposition, ultimately leading to fibrosis in patients with asthma and COPD (4, 5).

Patients with chronic respiratory diseases are more susceptible to viral infections and contribute to disease exacerbation and progression due to an exaggerated immune response (6). Most viral responses are regulated by innate immune mechanisms through the activation of Toll-like receptors (TLRs) (7). Although several TLRs are activated in antiviral infections, TLR3 activation is triggered by double-stranded (ds) RNA motifs, a viral replication intermediate that is produced by positive-single-stranded RNA viruses (8, 9). TLR3 is located intracellularly, attached to the membrane of endosomes, and expressed in many cell types, including airway smooth muscle cells (10). After binding the dsRNA motif, TLR3 dimerizes and recruits the TIR-domain-containing adapter-inducing interferon-β (TRIF) protein (11) to the endosome, which results in activation of pro-inflammatory transcription factors, namely interferon regulatory factors (IRFs), and nuclear factor κappa B (NF-κB) (9, 12). TLR3 signaling *via* TRIF results in the upregulation of multiple pro-inflammatory cytokines, such as interleukin-6 (*IL-6*), *IL-1β*, tumor necrosis factor-alpha (*TNF-α*) and chemokines. The chemokine (CXC motif) ligand 10 (*CXCL10*) and the chemokine (CC motif) ligand 2 (*CCL2*), encoding monocyte chemoattractant protein 1 (MCP-1), are potent inflammatory mediators involved in inflammatory immune cell migration. These pro-inflammatory mediators have been recently described in the context of viral infections (13) and activates T helper 1 (Th1) cell-mediated immune responses. Increases in pro-inflammatory mediators in viral infections, in turn, may further activate signaling pathways involved in fibrosis (14), characterized by excessive deposition of ECM proteins, mostly fibronectin 1 (FN1) and type I collagen (COL1A1) (15).

Several clinical studies have associated vitamin D deficiency with increased risk of pathogenesis in patients with chronic lung diseases, such asthma and COPD (16–18). Moreover, vitamin D3 (1,25D3) has been shown to modulate innate immune responses in various cell types, including BSMCs (19), through its binding to vitamin D receptors (VDRs) that downregulate several pro-inflammatory transcription factors activated during infections (20, 21). In addition, vitamin D3 influences the expression and activity of various TLRs (20), essential in the immune response against viral infections (22).

Although TLR3 has a major role in viral-induced immune responses, excessive TLR3 inflammatory responses may play a key role in promoting exacerbations and fibrosis in asthma and COPD. Therefore, in this study, we investigated the implications of vitamin D3 in offsetting pro-inflammatory and pro-fibrotic responses induced by TLR3 agonist, polyI:C in BSMCs isolated from asthmatic, COPD, and healthy control (non-smokers and smokers) subjects. We hypothesized that 1,25D3 treatment downregulates polyI:C-induced pro-inflammatory and pro-fibrotic responses in BSMCs. We assessed the mRNA expression of *TLR3*, *CYP24A1* and *VDR*, as a possible mechanism involved in these responses. Subsequently, we determined the mRNA expression and protein levels of selected markers (IL-6, IFN-β1, CCL2, fibronectin 1 and type I collagen) before and after exposing the BSMCs to polyI:C, either alone or combined with 1,25D3 treatment. Our results suggest that vitamin D3 may play a protective role in the development of exacerbations due to viral infections, by its ability to regulate TLR3 responses in BSMCs.

## Materials and Methods

### Human Sample Characteristics

For asthma experiments, Bronchial Smooth Muscle Cells (BSMCs) from non-asthmatic (n=3) and asthmatic (n=4) subjects were purchased from a commercial source (Lonza, MD, United States). These cells were chosen from subjects who were age-matched and non-smokers. For COPD experiments, BSMCs were obtained from the Hospital Research Institute of the University of Manitoba, as previously described (23). All procedures were approved by the Human Research Ethics Board of the University of Manitoba. These cells were chosen from subjects who were age-matched, smokers non-COPD (n=3) and smokers COPD (n=4). **Table 1** describes the characteristics of the subjects included in this study.

**Table 1:** Characteristics of the subjects included in this study.

Subjects’ characteristics	Asthma		COPD	
Groups	Non-asthmatics healthy controls	Asthma	Non-COPD healthy controls	COPD
Subjects, N	3	4	3	4
Age, years	37.8 ± 9.9	35 ± 4.4	74 ± 7	71.7 ± 4.8
Disease severity	–	severe	–	mild
Smoking status	non-smokers	non-smokers	smokers	smokers
Pack years of smoking	–	–	24.2 ± 2.4	36.3 ± 3.1
Gender, M/F	2/1	1/3	2/1	2/2
FEV1, %	ND	ND	109 ± 15.3	54.7 ± 14.7
FEV1/FVC ratio, %	ND	ND	82.8 ± 7.9	61.4 ± 7.4
Medication	none	albuterol, nasal steroids	none	albuterol, nasal steroids

FEV1, Forced Expiratory Volume at 1s; FVC, Forced vital capacity; M, male; F, female; ND, not determined. Values are mean ± SE. Pack years of smoking were calculated by multiplying the number of years smoked by the average number of packs per day.

### BSMCs Culture and Treatment

BSMCs from passages two to five were maintained in Dulbecco’s Modified Eagle’s Medium (DMEM) (Gibco-BRL Inc. Carlsbad, USA) supplemented with 10% Fetal Bovine Serum (FBS) and 1% Penicillin-Streptomycin in a humidified 5% CO_2_/37˚C incubator. For experiments, the cells were seeded into 12- or 6-well plates at a cellular density of 1 x 10^5^ cells and 2 x 10^5^ cells, respectively. At ~ 80% confluence, the cells were serum-starved overnight in FBS-free DMEM. BSMCs were then stimulated for 24 hours with 5 µg/ml of polyinosinic:polycytidylic acid (polyI:C) (R&D Systems, Minneapolis, USA) alone or combined with 1,25D3 (100 nM) (Sigma-Aldrich Int, USA) treatment. Both reagents were added simultaneously to the cells. Ethyl alcohol (EtOH) (0.1%) vehicle was added to unstimulated BSMCs and used as control for the experiments. After stimulation, the cells were processed for RNA extraction and cell-free culture media were collected and frozen for ELISA experiments.

### Quantitative Reverse Transcription-Polymerase Chain Reaction (qRT-PCR)

Gene expression levels of VDR, CYP24A1, TLR3, IL-6, IFN-β1, FN1, COL1A1, and CCL2 were determined in BMSCs groups: asthma versus healthy controls and smokers-COPD versus smokers-healthy controls by quantitative real time-PCR (qRT-PCR). Real time-PCR primers are listed in **Table 2**. Briefly, total RNA from BSMCs lysates was extracted using RiboZol reagent (VWR, Leicestershire, UK), as directed by the manufacturer’s instructions. Contaminating DNA was removed from 1 μg of total RNA using the AccuRT Genomic DNA Removal Kit and reverse transcribed into cDNA using the 5X All-In-One Reverse Transcriptase Master mix, according to the manufacturer’s instructions [Applied Biological Materials (abm), Richmond, BC, Canada]. Relative levels of targeted pro-inflammatory and pro-fibrotic genes’ mRNA were measured using BrightGreen qRT-PCR Master mix (abm). The qRT-PCR amplification was performed using CFX96 thermal cycler (BioRad, Hercules, USA) according to the manufacturer’s protocol (abm, BC, Canada). The gene-specific mRNA primer sequences listed in **Table 2** were designed using NCBI Primer3/BLAST software and synthesized by Life Technologies (Invitrogen). Each sample was tested in duplicates and the experiment was performed at least twice. The 2^-ΔΔCT^ method was applied to determine the relative fold change in gene expressions in samples treated with polyI:C alone or polyI:C-1,25D3-stimulated BSMCs from asthma/COPD relative to unstimulated BSMCs from healthy controls, after normalization to the housekeeping gene, Glyceraldehyde 3-phosphate Dehydrogenase (*GAPDH*), like previously described (24).

**Table 2:** Gene specific primer sequences used for qRT-PCR.

Gene	NCBI Reference	Forward primer (*5’ to 3’*)	Reverse primer (*5’ to 3’*)	Amplicon (bp)
*GAPDH*	NM_002046	GAAGGTGAAGGTCGGAGT	GAAGATGGTGATGGGATTTC	226
*VDR*	NM_000376	CTTCAGGCGAAGCATGAAGC	CCACCATCATTCACACGAACTGG	128
*CYP24A1*	NM_000782	GCTTCTCCAGAAGAATGCAGGG	CAGACCTTGGTGTTGAGGCTCT	125
*TLR3*	NM_003265	GCGCTAAAAAGTGAAGAACTGGAT	GCTGGACATTGTTCAGAAAGAGG	145
*IL-6*	NM_000600	ACCTTCCAAAGATGGCTGAAA	GCTCTGGCTTGTTCCCTCACTAC	153
*IFN-β1*	NM_002176	CTTGGATTCCTACAAAGAAGCAGC	TCCTCCTTCTGGAACTGCTGCA	146
*FN1*	NM_212482	CCAACTGGTAACCCTTCC	CCAACACTGGGTTGCTGA	156
*COL1A1*	NM_000088	GATTCCCTGGACCTAAAGGTGC	TCCAGCCTCTCCATCTTTGC	110
*CCL2*	NM_002982	CCCCAGTCACCTGCTGTTAT	TGGAATCCTGAACCCACTTC	171

GAPDH, Glyceraldehyde 3-phosphate dehydrogenase; VDR, Vitamin D receptor; CYP24A1, cytochrome P450 family 24 subfamily A member 1; TLR3, Toll like receptor 3; IL-6, Interleukin -6; IFN-β1, Interferon beta 1; FN1, Fibronectin 1; COL1A1, Collagen type I alpha chain 1; CCL2, Chemokine ligand 2.

### Quantification of Pro-Inflammatory and Pro-Fibrotic Markers

Cell-free supernatants from polyI:C alone or polyI:C-1,25D3-stimulated BSMCs were harvested and stored for further cytokine measurements. The following ELISA kits: Human CCL2/MCP-1 DuoSet DY279-05 (R&D Systems), Human Fibronectin (FN1) DuoSet ELISA DY1918-05 (R&D Systems), Human IFN-beta DuoSet ELISA DY814-05 (R&D Systems), and Human IL-6 ELISA MAX Deluxe (Cat. No. 430504, BioLegend) were used. Assay procedure was followed according to the manufacturer’s protocol. The limits of detection for all ELISAs’ kits were in picogram range (> 7.8 pg/ml), except for fibronectin 1 for which the limit of detection was > 0.1 ng/ml. The absorbance was read at 450 nm (corrections 570 nm) in a microplate reader (Epoch Spectrophotometer System).

### Quantification of Intracellular Type I Collagen in BSMCs

To quantify intracellular type I collagen produced in BSMCs, the anti-human type I collagen antibodies conjugated to fluorescein isothiocyanate (FITC) (Millipore, MAB3262F) and Live/Dead Fixable Violet (ThermoFisher Scientific, L34955) dual staining was performed, according to the manufacturer`s instructions. Briefly, polyI:C alone and polyI:C-1,25D3-stimulated BSMCs were harvested using non-enzymatic cell stripper solution (Corning, Manassas, VA, USA), centrifuged for 5 min at 500xg and 2 x 10^5^ cells were pre-incubated with Live/Dead dye (1/1000) for 15 minutes at room temperature. Cells were then washed with phosphate-buffered saline (PBS) and fixed with 2% paraformaldehyde (PFA) for 10 minutes. After PBS washing, the cells were immunolabelled for 1 h at room temperature with anti-human type I collagen-FITC (1/200) diluted in permeabilization buffer (0.05% Triton, 1% BSA in PBS). Then, the cells were washed with PBS and kept on ice while they were analyzed by flow cytometry (FACSCanto II, BD Biosciences, USA). EtOH (0.1%) treated and unstained cells served as control samples. Compensation beads (Invitrogen, Ref. 01-2222-41) were stained with anti-human type I collagen-FITC or Pacific Blue Mouse IgG1 isotype control (BD Pharmingen, Cat. 558120) and used as compensation controls, according to the manufacturer`s instructions. For each sample, 1×10^5^ single cell events were acquired and analyzed by FlowJo 10.0.7 software. Live-cells gating strategy to analyze type I collagen positive cells is shown in **Figures 6E–G**.

### Statistical Analysis

One-way analysis of variance (ANOVA) coupled with Newman‐Keuls post-hoc tests were performed to assess statistical significance between groups. All results are presented as mean ± standard error (SE) from 2 independent experiments using GraphPad Prism 5 (GraphPad, San Diego, CA, USA). A *p* value > 0.05 was considered not statistically significant (*ns*). The level of significance was set at *p ≤ 0.05, **p < 0.01, and ***p < 0.001.

## Results

### BSMCs Respond to 1,25D3 by Inducing the Expression of *CYP24A1*, Regulated by VDR

**Figure 1:**
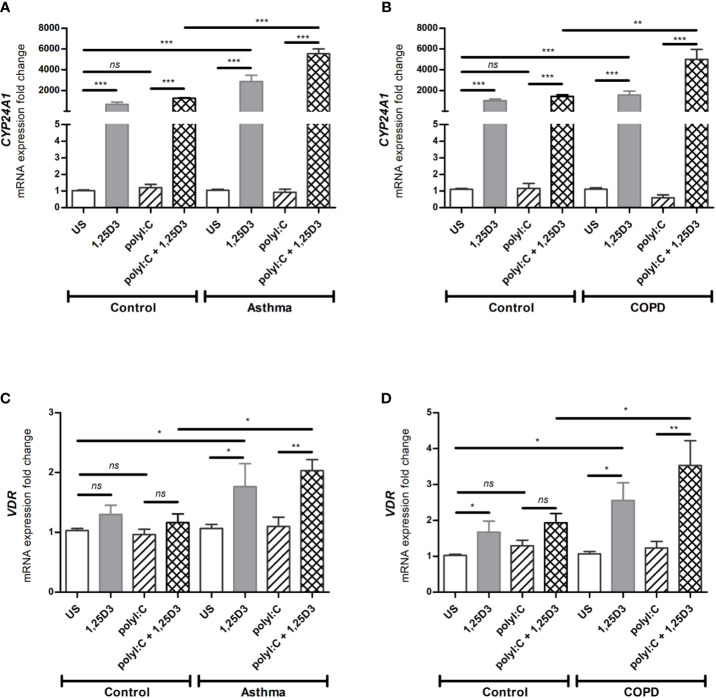
mRNA expression of *CYP24A1* **(A, B)** and *VDR* **(C, D)** in BSMCs from asthma **(A, C)** and COPD **(B, D)** compared to BSMCs from healthy control groups. *n* = 4 asthma, *n* = 3 healthy controls, *n* = 4 COPD, and *n* = 3 healthy control smokers. qRT-PCR data is representative of two independent experiments. One way ANOVA using Newman-Keuls multiple comparison test were performed to assess statistical significance between groups. Mean ± SE; (*ns*) *p* > 0.05, no significant difference, *p < 0.05, **p < 0.01, ***p < 0.001.

To determine whether BSMCs respond functionally to 1,25D3 treatment, the relative expression of *CYP24A1* and *VDR* were tested by qRT-PCR in polyI:C-stimulated BSMCs, with or without 1,25D3 for 24 hours. *CYP24A1* gene expression was significantly upregulated upon addition of 1,25D3 to polyI:C-stimulated BSMCs, while polyI:C alone had no significant effect. The addition of 1,25D3 to polyI:C-stimulated BSMCs significantly induced *CYP24A1* mRNA expression in asthma (5547 ± 454-fold increase, *p* < 0.001) (**Figure 1A**) and COPD (3565 ± 311-fold increase, *p* < 0.01) (**Figure 1B**) as compared to control groups. The mRNA expression of *VDR* was only slightly increased in polyI:C-stimulated BSMCs and this effect was significantly increased by the addition of 1,25D3 (0.866 ± 0.23-fold increase, *p* = 0.01 in asthma (**Figure 1C**) and 1.6 ± 0.73-fold increase in COPD, *p* < 0.05) (**Figure 1D**). The effect of 1,25D3 treatment alone in BSMCs was also investigated. We observed statistically significant increase in mRNA *CYP24A1* and *VDR* expression (*p* < 0.05) when 1,25D3 alone was added to the cells and this effect was observed at a higher extent in diseased BSMCs as compared to non-diseased BSMCs, (**Figures 1A–D**).

### 1,25D3 Decreases the Expression of *TLR3* Activated in Response to PolyI:C

**Figure 2:**
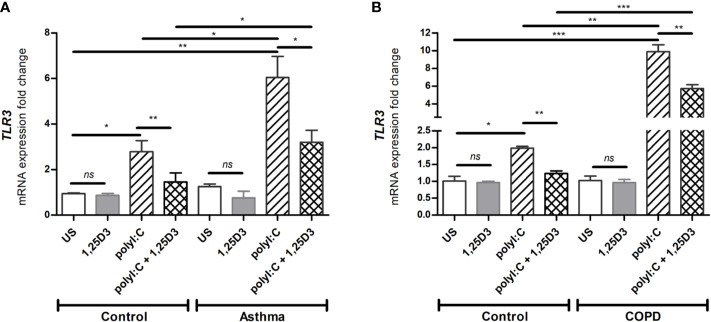
mRNA expression of *TLR3* in asthma **(A)** and COPD **(B)** compared to healthy control groups. *n* = 4 asthma, *n* = 3 healthy controls, *n* = 4 COPD, and *n* = 3 healthy control smokers. qRT-PCR data is representative of two independent experiments. One way ANOVA using Newman-Keuls multiple comparison test were performed to assess statistical significance between groups. Mean ± SE; (*ns*) *p* > 0.05, no significant difference, *p < 0.05, **p < 0.01, ***p < 0.001.

Since TLR3 activation is required for polyI:C-induced pro-inflammatory and pro-fibrotic mediators release, we further investigated whether 1,25D3 treatment influences *TLR3* expression in BSMCs. Stimulation of BSMCs with polyI:C (5 ug/ml) for 24 hours significantly induced mRNA expression of *TLR3*, in asthma (6.047 ± 0.924-fold increase, *p* < 0.05) (**Figure 2A**) and COPD (9.878 ± 0.779-fold increase, *p* < 0.001) (**Figure 2B**) as compared to control groups. While Addition of 1,25D3 to polyI:C-stimulated BSMCs significantly decreased *TLR3* expression, in asthma (1.743 ± 0.6387-fold decrease, *p* < 0.05) (**Figure 2A**) and COPD (4.495 ± 0.6318-fold decrease, *p* < 0.05) (**Figure 2B**) as compared to control groups. On the contrary, 1,25D3 treatment alone had no statistically significant effect (*p* > 0.05) on *TLR3* mRNA expression in BSMCs, (**Figures 2A, B**).

### 1,25D3 Decreases PolyI:C-Induced Release of Pro-Inflammatory and Pro-Fibrotic Markers in BSMCs

**Figure 3:**
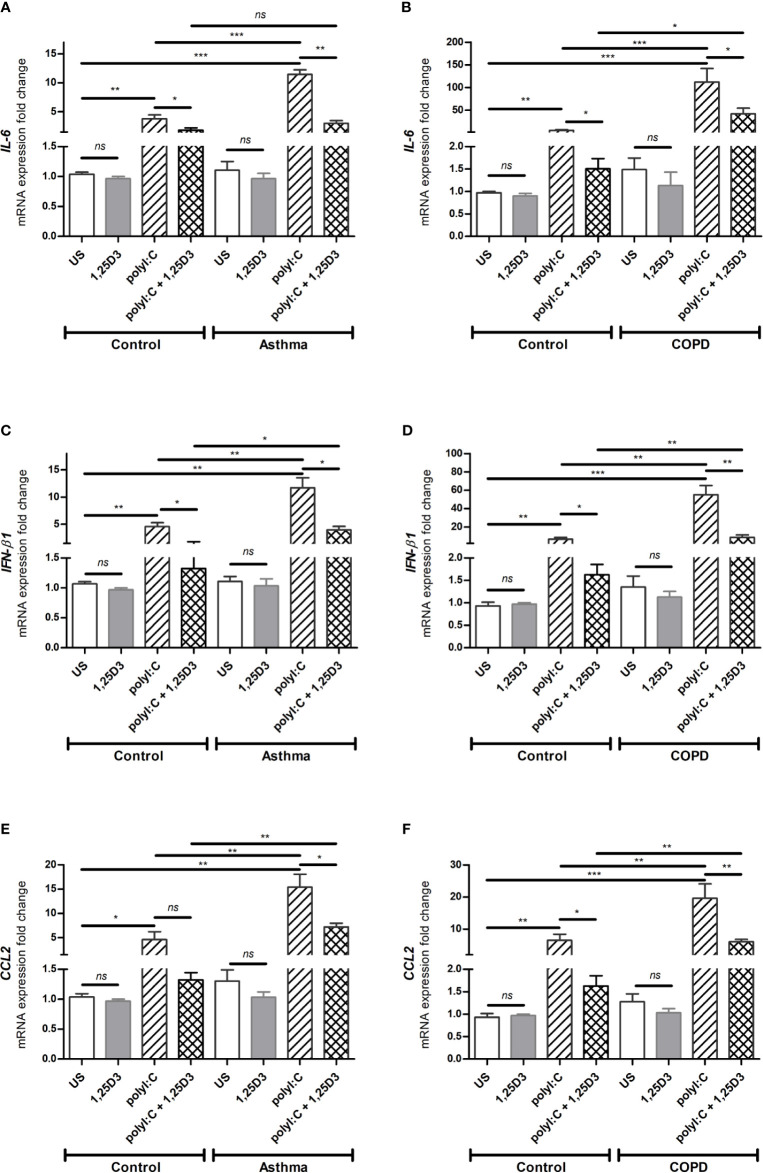
mRNA expression of *IL-6* **(A, B)**, *IFN-β1* **(C, D)** and *CCL2* **(E, F)** in BSMCs from severe asthma **(A, C, E)** and mild COPD **(B, D, F)** compared to BSMCs from healthy control groups. *n* = 4 asthma, *n* = 3 healthy controls, *n* = 4 COPD, and *n* = 3 healthy control smokers. qRT-PCR data is representative of two independent experiments. One way ANOVA using Newman-Keuls multiple comparison test were performed to assess statistical significance between groups. Mean ± SE; (*ns*) *p* > 0.05, no significant difference, *p < 0.05, **p < 0.01, ***p < 0.001.

Based on our dose-response experiments (**data not shown**), polyI:C at 5 µg/ml was considered optimal and used to determine the pro-inflammatory and pro-fibrotic responses in BSMCs. Because 1,25D3 has anti-inflammatory and anti-fibrotic effects, we hypothesized that 1,25D3 treatment decreases the pro-inflammatory and pro-fibrotic responses in polyI:C-stimulated BSMCs. Therefore, BSMCs were stimulated with polyI:C (5 µg/ml) alone or in combination with 1,25D3 (100 nM) for 24 hours. Following stimulation, BSMCs were collected, and RNA was extracted. As shown in **Figures 3A–F**, BSMCs treated with polyI:C, had a significant increase in mRNA expression of *IL-6*, *IFN-β1*, *CCL2* compared to untreated cells and this effect was observed to a higher extent in asthma and COPD BSMCs (*p* < 0.05). When 1,25D3 was added to polyI:C-stimulated BSMCs, there was a significant decrease in mRNA expression of *IL-6*, *IFN-β1*, *CCL2*. In addition, we observed a higher extent of the anti-inflammatory effect of 1,25D3 in BSMCs from COPD, namely for *IL-6* (40.24 ± 15.39-fold decrease, *p* < 0.05, **Figure 3B**) and *IFN-β1* (6.65 ± 2.21-fold decrease, *p* < 0.01, **Figure 3D**), than in BSMCs from asthma (**Figures 3A, C**, **Table S1A**). The intra- and inter-group relative fold change differences in the expression of pro-inflammatory and pro-fibrotic markers among groups are described in the **Supplementary Data** (**Tables S1A **and** B**).

**Figure 4:**
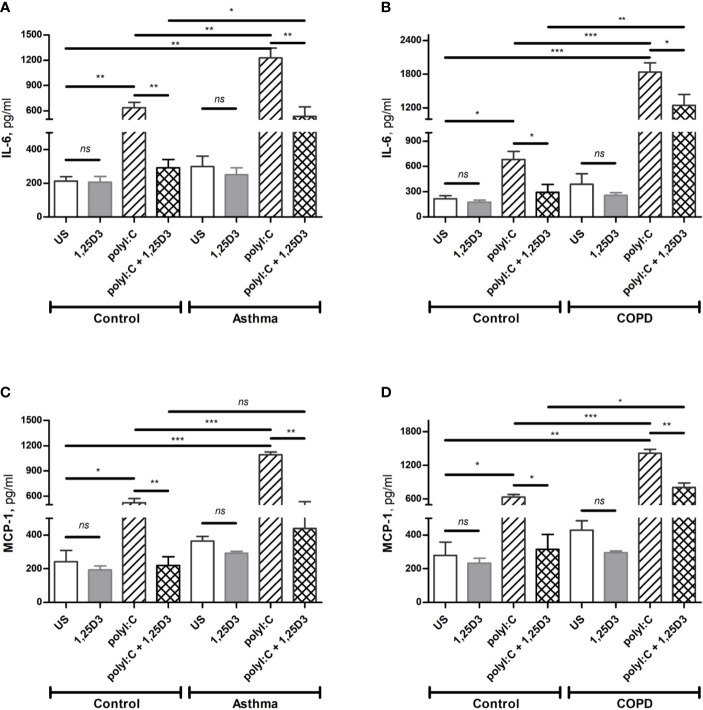
Protein levels of IL-6 **(A, B)**, and MCP-1 **(C, D)** in BSMCs from asthma **(A, C)** and COPD **(B, D)** compared to BSMCs from healthy control groups were quantified by ELISA. *n* = 4 asthma, *n* = 3 healthy controls, *n* = 4 COPD, and *n* = 3 healthy control smokers. Data is representative of two independent experiments. One way ANOVA using Newman-Keuls multiple comparison test were performed to assess statistical significance between groups. Mean ± SE; (*ns*) *p* > 0.05, no significant difference, *p < 0.05, **p < 0.01, ***p < 0.001.

To confirm these findings, ELISA was performed on conditioned media obtained from BSMCs stimulated with polyI:C or polyI:C-1,25D25, and protein levels of IL-6, IFN-β1 and MCP-1 were assessed. Similarly, polyI:C stimulation significantly increased IL-6 and MCP-1 protein levels in asthmatic and COPD compared to control groups (**Figures 4A–D** and **Table S1A**). A significant overall decrease in the protein levels of IL-6 and MCP-1 (**Figures 4A–D** and **Table S1B**) was detected upon the addition of 1,25D3 to polyI:C-stimulated BSMCs. Furthermore, an increased anti-inflammatory effect of 1,25D3 was observed in BSMCs from COPD, for secreted IL-6 (954 ± 217-fold decrease, *p* < 0.001, **Figure 4B**) and MCP-1 (488.9 ± 120-fold decrease, *p* < 0.01, **Figure 4D** and **Table S1A**) than in BSMCs from asthma (**Figures 4A, C** and **Table S1A**). However, we could not validate the mRNA data for IFN-β1 by ELISA assay, since the protein levels of IFN-β1 were under the detection limit (< 7.8 pg/ml) (data not shown).

**Figure 5:**
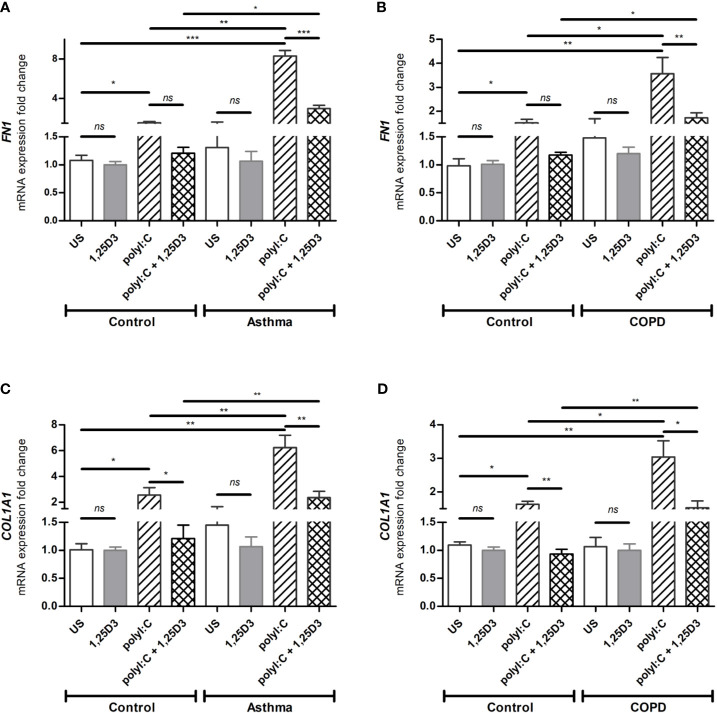
mRNA expression of *FN1* **(A, B)** and *COL1A1* **(C, D)** in BSMCs from asthma **(A, C)** and COPD **(B, D)** compared to BSMCs from healthy control groups. *n* = 4 asthma, *n* = 3 healthy controls, *n* = 4 COPD, and *n* = 3 healthy control smokers. qRT-PCR data is representative of two independent experiments. One way ANOVA using Newman-Keuls multiple comparison test were performed to assess statistical significance between groups. Mean ± SE; (*ns*) *p* > 0.05, no significant difference, *p < 0.05, **p < 0.01, ***p < 0.001.

**Figure 6:**
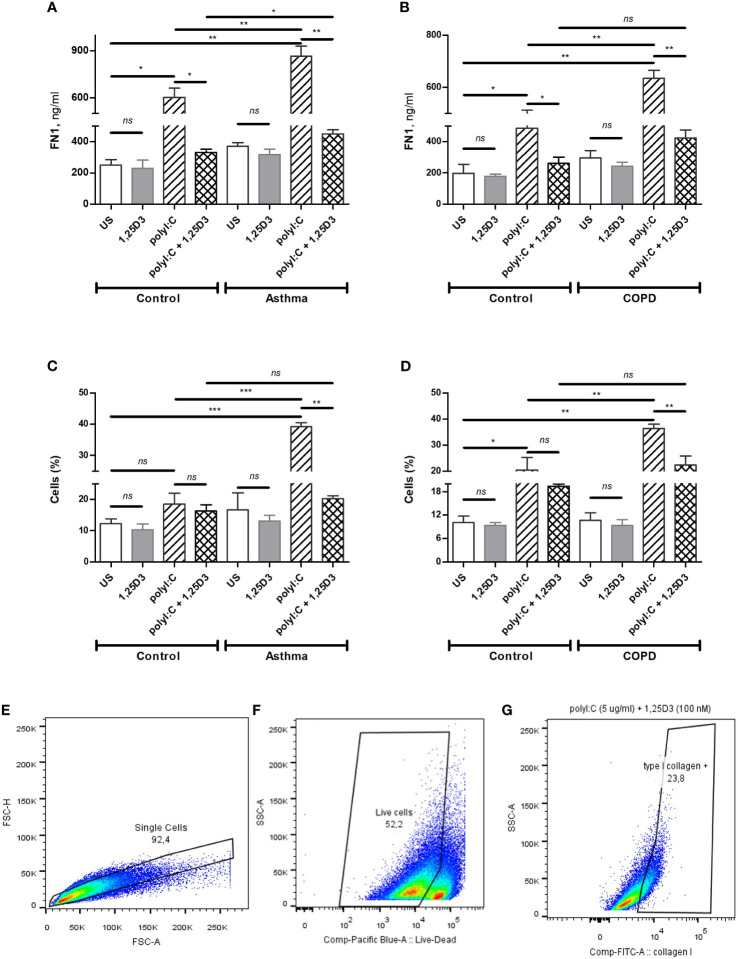
Protein levels of FN1 in BSMCs from asthma **(A)** and COPD **(B)** compared to BSMCs from healthy control groups were quantified by ELISA. Flow Cytometry was used to quantify intracellular levels of type I collagen in BSMCs from asthma **(C)** and COPD **(D)** groups. Graphic quantitation, % of type I collagen positive BSMCs populations using GraphPad **(C, D)**. Gating strategy of type I collagen positive BSMCs: **(E)** Exclusion of doublet events. **(F)** Exclusion of dead cells. **(G)** Gating of type I collagen positive BSMCs. The number of cells (%) included in each gate is indicated in each panel. *n* = 4 asthma, *n* = 3 healthy controls, *n* = 4 COPD, and *n* = 3 healthy control smokers. Data is representative of two independent experiments. One way ANOVA using Newman-Keuls multiple comparison test were performed to assess statistical significance between groups. Mean ± SE; (*ns*) *p* > 0.05, no significant difference, *p < 0.05, **p < 0.01, ***p < 0.001.

To test whether TLR3 activation might control the expression of pro-fibrotic genes, mRNA expression of *FN1* and *COL1A1* were assessed in polyI:C alone or polyI:C-1,25D3-stimulated BSMCs. BSMCs treated with polyI:C alone displayed an increase in the mRNA expression of *FN1* and *COL1A1* compared to untreated cells (**Figures 5A–D**), and this effect was observed to a higher extent in asthma and COPD as compared to control groups (*p* < 0.05) (**Figures 5A–D** and **Table S1A**). 1,25D3 treatment significantly decreased polyI:C-induced mRNA expression of *FN1* and *COL1A1*, as described in **Table S1B**. Increased anti-fibrotic effect of 1,25D3 was observed in BSMCs from asthma (**Figures 5A, C** and **Table S1A**; *FN1* average fold-decrease 6.78 ± 0.65, *p* < 0.001; *COL1A1* average fold-decrease 3.68 ± 1.21, *p* < 0.05 *p* = 0.014) compared to BSMCs from COPD (**Figures 5B, D** and **Table S1A**). Similarly, 1,25D3 treatment significantly decreased protein levels of FN1 and mean number of the type I collagen positive cells in polyI:C-stimulated BSMCs, to a greater extent in asthma (**Figures 6A, C** and **Table S1A**; fibronectin 1 average fold-decrease 263 ± 94, *p* < 0.05; type I collagen average fold-decrease 20.67 ± 3.77 *p* < 0.01) than in COPD (**Figures 6B, D** and **Table S1A**. While an overall decrease in mRNA expression and protein levels of pro-inflammatory (**Figures 3A–F** and **4A–D**) and pro-fibrotic markers (**Figures 5A–D** and **6A–D**) was observed upon the addition of 1,25D3 alone to BSMCs, the effect observed was not statistically significant (*p* > 0.05). Although not statistically significant, we observed an increased overall mRNA expression of pro-inflammatory and pro-fibrotic markers at the baseline in unstimulated-BSMCs from asthma (**Figures 3A, C, E** and **5A, C**) and COPD (**Figures 3B, D, F** and **5B, D**) compared to unstimulated-BSMCs from healthy control groups. Similarly, we observed an increased overall protein level of pro-inflammatory and pro-fibrotic markers at the baseline in unstimulated-BSMCs from asthma (**Figures 4A, C** and **6A, C**) and COPD (**Figures 4B, C** and **6B, D**) compared to unstimulated-BSMCs from healthy control groups.

## Discussion

Abnormal immune responses in viral infections may worsen acute lung injury, increasing airflow obstruction or pulmonary fibrosis, and pathological conditions already existent in patients with asthma or COPD.

In the present study, we have demonstrated the effect of vitamin D3 supplementation on viral-induced TLR3 responses in Bronchial Smooth Muscle Cells (BSMCs), as a mechanism contributing to pulmonary fibrosis in asthma and COPD. Polyinosinic: polycytidylic acid (polyI:C), a synthetic analog of viral double-stranded RNA, has been previously described in driving viral immunomodulatory response (25) and dermal fibrosis (14) in cultured fibroblasts through the activation of TLR3 receptors. Although fibroblasts are the major contributors to airway remodeling in asthma and COPD, other cell types within the lung, including smooth muscle cells are also involved. On the other hand, vitamin D3 through its active metabolite 1,25D3, is known to possess anti-inflammatory and anti-fibrotic mechanisms (26–28), and have shown to improve immune responses (29, 30). Therefore, we investigated the role of 1,25D3 in polyI:C-induced pro-inflammatory and pro-fibrotic responses in primary BSMCs isolated from subjects with asthma and COPD. The BSMCs used in this study were age-matched for asthma experiments, while for COPD experiments, in addition to age, smoking history was also considered. Cigarette smoke, a highly prevalent risk factor in COPD, is also known to alter the expression and function of TLR3 receptors (31). Our findings indicated that pro-inflammatory and pro-fibrotic mediators are increased at the baseline in BSMCs from both asthma and COPD, and that TLR3 agonist polyI:C, significantly upregulated *IL-6*, *IFN-β1*, *CCL2*, *FN1* and *COL1A1* expressions in BSMCs (**Figures 3A–F** and **5A–D**). The selected markers in this study represent pro-inflammatory and pro-fibrotic gene signatures in both viral infections, and in chronic respiratory diseases. Our findings were further supported by demonstrating the significant downregulation of these markers upon 1,25D3, in polyI:C-stimulated BSMCs, in the context of asthma and COPD when compared to control groups.

To gain a mechanistic insight into the effect of vitamin D3 on the activation and functionality of vitamin D receptors (VDR) in BSMCs, the effects of 1,25D3 supplementation of polyI:C-stimulated BSMCs was investigated. We observed a significant increase in the mRNA expression of *CYP24A1* when 1,25D3 was added to the polyI:C stimulated BSMCs, whereas polyI:C alone slightly altered its mRNA expression (**Figures 1A, B**). Cytochrome P450 family 24 subfamily A member 1 (*CYP24A1*) encodes 24-hydroxylase, an enzyme that regulates vitamin D metabolism through a negative feedback loop activation, thereby regulating its own metabolism (32). This result was therefore expected since *CYP24A1* is highly upregulated by 1,25D3 through a VDR-dependent mechanism. The mRNA expression of *VDR* was also increased when 1,25D3 was added to polyI:C stimulated BSMCs (**Figure 1C, D**). Moreover, our data revealed an increased mRNA expression of *TLR3* in polyI:C-stimulated BSMCs, and this effect was significantly diminished after 1,25D3 treatment (**Figures 2A, B**). Interestingly, we observed a higher grade of *VDR* and *TLR3* activation in BSMCs from subjects with asthma and COPD when compared with controls (**Figures 1C, D** and **2A, B**). These data suggest that BSMCs express functional *VDR* and *TLR3*, and that BSMCs from diseased groups (asthma and COPD) may have increased sensitivity to polyI:C than BSMCs from control groups.

Previous studies have shown that BSMCs express TLRs (10, 33), including *TLR3*, which may indicate their ability to respond to innate immune stimuli and a possible role in viral-induced inflammatory exacerbations. Although TLR3 agonists are well known to stimulate type I interferons (*IFN-α* and *IFN-β1*), their activation also results in upregulation of a variety of NF-kB regulated pro-inflammatory cytokines and chemokines, including *IL-6*, *IL-8*, tumor necrosis factor alpha (*TNF-α*) and *CCL2* (34). Our data also demonstrated an increased mRNA expression of *IL-6*, *IFN-β1* and *CCL2* in polyI:C-stimulated BSMCs from diseased groups when compared with controls (**Figures 3A–F**), and this increase was observed to a significantly greater extent in COPD cells (**Figures 3B, D, F**). Moreover, cell culture media obtained from polyI:C-stimulated BSMCs showed increased IL-6 and MCP-1 protein secretion, while 1,25D3 treatment significantly attenuated their levels (**Figures 4A–D**). While we noticed, prior to stimulation, an increased baseline level of these pro-inflammatory cytokines in BSMCs from diseased groups, the effect observed was not statistically significant. These data suggest that BSMCs from asthma and COPD are more prone to develop an inflammatory and fibrotic phenotype than the controls. These data suggest that polyI:C-stimulation leads to enhanced inflammatory responses in BSMCs from diseased groups, and that 1,25D3 acts on TLR3 to modulate the pro-inflammatory responses in polyI:C-stimulated BSMCs. Our data also points towards a more effective anti-inflammatory effect of 1,25D3 in polyI:C-stimulated BSMCs from subjects with COPD when compared to asthma (**Table S1A**). This pattern in response to 1,25D3 treatment could be beneficial, particularly during the initial stage of viral infection, therefore limiting the amount of pro-inflammatory mediators and protecting the lung tissue from further damage. Interestingly, we observed a lack of IFN-β1 secretion (as measured by ELISA, data not shown) and this result was unpredicted since the mRNA expression of *IFN-β1* was highly upregulated in polyI:C-stimulation BSMCs (**Figures 3C, D**). This is supported by a previous study, where Mazaleuskaya et al. provided evidence of high expression levels of *IFN-β1* in polyI:C-stimulated murine macrophages at 24 h, but the secreted IFN-β1 was only detected at 6 h post stimulation (25). Taken together, these results suggest that IFN-β1 response in cells is regulated differentially in time post transcriptionally and that the levels of IFN-β1 should be determined at shorter intervals than 24 h post-stimulation. In agreement with this, another study mentioned negative regulation of IFN-β1 production through transcriptional inactivation of IRF3, which may play a protective role reducing exaggerated inflammatory immune responses and limiting the duration of IFN-β1 activation in the host cells during persistent virus infection (35).

In addition, we aimed to establish whether polyI:C-stimulation of BSMCs also increased the mRNA expression and protein levels of FN1 and type I collagen, two pro-fibrotic mediators highly expressed in the airways of asthma and COPD patients. The mechanism by which viral infections cause lung fibrosis is not fully understood. It has been suggested that multiple inflammatory pathways are activated during viral infections, which interplay with the major contributors in lung fibrosis, such as transforming growth factor beta (TGF-β) Smad signaling, and the ECM turnover mechanisms in asthma and COPD (14, 36). Our data showed, prior to polyI:C stimulation, an increased basal expression of *FN1* and *COL1A1* in BSMCs from diseased groups, although this finding did not reach statistical significance. Following polyI:C-stimulation, the mRNA expression and protein levels of FN1 and COL1A1 were increased in BSMCs and 1,25D3 treatment significantly decreased their levels (**Figures 5A–D**). Interestingly, under polyI:C stimulation, BSMCs from subjects with asthma (**Figures 5A, C** and **Table S1A**) were more prone to a pro-fibrotic phenotype compared to BSMCs from COPD subjects (**Figures 5B, D** and **Table S1A**). Similarly, the level of fibronectin 1 and type I collagen was increased in polyI:C-stimulated BSMCs compared to unstimulated BSMCs, and 1,25D3 treatment significantly attenuated their levels (**Figures 6A–D**). Interestingly, 1,25D3 treatment alone showed limited effect on the expression and protein levels of pro-inflammatory and pro-fibrotic fibrotic markers in BSMCs without prior stimulation with polyI:C, as shown previously by other groups (22).

Study limitations. From the clinical data, patients with severe asthma or COPD are predisposed to severe lung injury and presented an increased risk of fibrosis compared to mild-to-moderate illnesses. The main limitation to this study is that BSMCs from COPD group were exclusively from subjects with mild COPD because of sample availability. However, it is also known that subjects with mild COPD presented underlying inflammation in the airways and are at increased risk of respiratory infections compared to healthy subjects (37, 38). Another limit of the study was that no available data on the vitamin D status or supplements or additional medication for the subjects included in this study, as this information is not available from the supplier.

Recent clinical evidence identified COPD and asthma as comorbidities in COVID-19 infections, and patients with severe COVID-19 infection have extensive pulmonary fibrotic tissue, in addition to an enhanced inflammatory state (39–41). Although TLR3 activation is triggered by double-stranded (ds) RNA motifs, produced during the replication of positive-single-stranded RNA viruses, such as SARS-CoV-2, there are no research studies to demonstrate converging pathways between SARS-CoV-2 receptor and PRRs.

In conclusion, our findings demonstrated that TLR3 agonist polyI:C induce pro-inflammatory and pro-fibrotic responses in BSMCs and suggest their potential for deregulation of the pathways involved in fibrotic responses. Moreover, we have demonstrated that 1,25D3 by VDR-TLR3 dependent pathways, significantly reduced the pro-inflammatory and pro-fibrotic effects in polyI:C-stimulated BSMCs, indicating a protective function of 1,25D3 in patients with chronic respiratory conditions and viral infections. Further research is required to shed the light on the interplay between SARS-CoV-2, PRRs and other lung cell types, in the context of 1,25D3 supplementation as a potential antiviral adjuvant therapy in COVID-19 patients with asthma and COPD.

## Data Availability Statement

All datasets generated for this study are included in the article/**Supplementary Material**.

## Ethics Statement

The studies involving human participants were reviewed and approved by Human Research Ethics Board of the University of Manitoba. The patients/participants provided their written informed consent to participate in this study.

## Author Contributions

MP performed experiments, analyzed the data, and drafted the manuscript. MG and AM contributed to data analysis and manuscript preparation. NJ participated in the revision of the manuscript. AH provided BSMCs from healthy smokers and COPD subjects and participated in the revision of the manuscript. SA advised throughout the study development and participated in the revision of the manuscript. QH contributed to the design of the experiments and participated in the revision of the manuscript. All authors contributed to the article and approved the submitted version.

## Funding

This research was funded by the Richard and Edith Strauss Foundation.

## Conflict of Interest

The authors declare that the research was conducted in the absence of any commercial or financial relationships that could be construed as a potential conflict of interest.

## Publisher’s Note

All claims expressed in this article are solely those of the authors and do not necessarily represent those of their affiliated organizations, or those of the publisher, the editors and the reviewers. Any product that may be evaluated in this article, or claim that may be made by its manufacturer, is not guaranteed or endorsed by the publisher.

## Supplementary Material

Supplementary Table 1A Inter-group comparisons: BSMCs from healthy controls versus BSMCs from asthma subjects; BSMCs from healthy control smokers versus BSMCs from COPD subjects. Mean difference in mRNA expression of *IL-6*, *IFN-β1*, *CCL2*, *FN1* and *COL1A1* was quantified by qRT-PCR. Cell-free culture media was used for protein quantification by ELISA for IL-6, MCP-1, and fibronectin 1. The intracellular quantification of type I collagen in BSMCs was performed by Flow cytometry. Data are represented as mean ± SE relative to the control and representative of two independent experiments. One way ANOVA using Newman-Keuls multiple comparison test were performed to assess statistical significance between groups. *p* > 0.05, no significant difference.

Supplementary Table 1B Intra-group comparisons: BSMCs from healthy controls and BSMCs from asthma subjects; BSMCs from healthy control smokers and BSMCs from COPD subjects. Mean difference in mRNA expression of *IL-6*, *IFN-β1*, *CCL2*, *FN1* and *COL1A1* was quantified by qRT-PCR. Cell-free culture media was used for protein quantification by ELISA for IL-6, MCP-1, and fibronectin 1. The intracellular quantification of type I collagen in BSMCs was performed by Flow cytometry. Data are represented as mean ± SE relative to the control and representative of two independent experiments. One way ANOVA using Newman-Keuls multiple comparison test were performed to assess statistical significance between groups. *p* > 0.05, no significant difference.
