# Supplementary material for: Vitamin D3 Attenuates Viral-Induced Inflammation and Fibrotic Responses in Bronchial Smooth Muscle Cells
Source: Front Immunol. 2021 Aug 26;12:715848. doi: 10.3389/fimmu.2021.715848 (PMC8427752; doi:10.3389/fimmu.2021.715848)
Supplement: Supplementary Table 1A — Inter-group comparisons: BSMCs from healthy controls versus BSMCs from asthma subjects; BSMCs from healthy control smokers versus BSMCs from COPD subjects. Mean difference in mRNA expression of IL-6, IFN-β1, CCL2, FN1 and COL1A1 was quantified by qRT-PCR. Cell-free culture media was used for protein quantification by ELISA for IL-6, MCP-1, and fibronectin 1. The intracellular quantification of type I collagen in BSMCs was performed by Flow cytometry. Data are represented as mean ± SE relative to the control and representative of two independent experiments. One way ANOVA using Newman-Keuls multiple comparison test were performed to assess statistical significance between groups. p > 0.05, no significant difference. [file Table_1.pdf]

TABLE S1A. Inter-group comparisons: BSMCs from healthy controls versus BSMCs from asthma subjects; BSMCs from healthy control smokers versus BSMCs from COPD subjects

Inter-group comparisons: BSMCs Healthy Controls versus Asthma				
Pro-inflammatory and pro-fibrotic markers	polyI:C-BSMCs Controls versus polyI:C-BSMCs Asthma		polyI:C+1,25D3-BSMCs Controls versus polyI:C + 1,25D3-BSMCs Asthma	
	**mRNA expression**		**mRNA expression**	
	mean difference ± SEM	*p* values	mean difference ± SEM	*p* values
IL-6	7.68 ± 1.08	< 0.001	1,16 ± 0.67	> 0.05
IFN-β1	7.08 ± 2.25	< 0.05	2.64 ± 0.85	< 0.05
CCL2	10.81 ± 3.44	< 0.05	5.88 ± 0.91	< 0.001
FN1	6.78 ± 0.65	< 0.01	1.77 ± 0.41	< 0.01
COL1A1	3.68 ± 1.21	< 0.05	2.73 ± 0.43	> 0.05
	**Protein levels**		**Protein levels**	
	mean difference ± SEM	*p* values	mean difference ± SEM	*p* values
**IL-6**	592.8 ± 132	< 0.01	241.6 ± 123	> 0.05
**MCP-1**	571.9 ± 358.5	< 0.001	219 ± 108	> 0.05
**FN1**	263 ± 94	< 0.05	118 ±40	< 0.05 0.033
**Type I collagen**	20.67 ± 3.77	< 0.01	3.97 ± 2.2	> 0.05

Inter-group comparisons: BSMCs Healthy Control Smokers versus COPD Smokers				
Pro-inflammatory and pro-fibrotic markers	polyI:C-BSMCs Controls versus polyI:C-BSMCs COPD		polyI:C+1,25D3-BSMCs Controls versus polyI:C + 1,25D3-BSMCs COPD	
	**mRNA expression**		**mRNA expression**	
	mean difference ± SEM	*p* values	mean difference ± SEM	*p* values
IL-6	13.12 ± 5.57	< 0.05	40.24 ± 15.39	< 0.05
IFN-β1	48.57 ± 12.12	< 0.01	6.65 ±2.21	< 0.01
CCL2	13.12 ± 5.57	< 0.05	5.3 ± 0.45	< 0.01
FN1	2.05 ± 0.8	< 0.05	0.54 ± 0.24	< 0.05
COL1A1	1.4 ± 0.57	< 0.05	0.59 ± 0.25	< 0.05 0.033
	**Protein levels**		**Protein levels**	
	mean difference ± SEM	*p* values	mean difference ± SEM	*p* values
**IL-6**	1150 ± 188	< 0.01	954 ± 217	< 0.001
**MCP-1**	784 ± 85.9	< 0.001	488.9 ± 120	< 0.01
**FN1**	181 ± 74	< 0.05	69.3 ± 58.73	> 0.05
**Type I collagen**	6.0 ± 2.52	< 0.05	2.93 ± 3.35	> 0.05

TABLE S1B. Intra-group comparisons: BSMCs from healthy controls and BSMCs from asthma subjects; BSMCs from healthy control smokers and BSMCs from COPD subjects

**Intra-group comparisons: BSMC -Healthy Controls and Asthma**				
Pro-inflammatory and pro-fibrotic markers	polyI:C, BSMCs Controls versus polyI:C + 1,25D3, BSMCs Controls		polyI:C, BSMCs Asthma versus polyI:C + 1,25D3, BSMCs Asthma	
	**mRNA expression**		**mRNA expression**	
	mean difference ± SEM	*p* values	mean difference ± SEM	*p* values
IL-6	1.95 ± 0.8	< 0.05	8.47 ± 0.91	< 0.001
IFN-β1	3.26 ± 0.82	< 0.01	7.71 ± 1.95	< 0.01
CCL2	3.39 ± 1.63	> 0.05	8.22 ± 2.78	< 0.05
FN1	0.32 ± 0.17	> 0.05	5.33 ± 0.64	< 0.0001
COL1A1	1.35 ± 0.62	< 0.05	3.88 ± 1.06	< 0.01
	**Protein levels**		**Protein levels**	
	mean difference ± SEM	*p* values	mean difference ± SEM	*p* values
**IL-6**	343.2 ± 80.9	< 0.01	694.4 ± 162	< 0.01
**MCP-1**	301.6 ± 71.1	< 0.01	653 ± 100	< 0.01
**FN1**	270 ± 62.9	< 0.05	416 ± 70.3	< 0.01
**Type I collagen**	4.03 ± 2.2	> 0.05	18.9 ± 1.69	< 0.001

**Intra-group comparisons: BSMCs Healthy Control Smokers and COPD Smokers**				
Pro-inflammatory and pro-fibrotic markers	polyI:C, BSMCs Controls versus polyI:C + 1,25D3, BSMCs Controls		polyI:C, BSMCs COPD versus polyI:C + 1,25D3, BSMCs COPD	
	**mRNA expression**		**mRNA expression**	
	mean difference ± SEM	*p* values	mean difference ± SEM	*p* values
IL-6	3.63 ± 1.77	< 0.05	70.3 ±32.66	< 0.05
IFN-β1	5.14 ± 1.61	< 0.05	46.61 ± 10.52	0.0022
CCL2	4.84 ± 1.9	< 0.05	13.53 ± 4.57	< 0.05
FN1	0.3 ± 0.15	> 0.05	1.84 ± 0.71	< 0.05
COL1A1	0.7 ± 0.12	< 0.01	1.51 ± 0.52	< 0.05
	**Protein levels**		**Protein levels**	
	mean difference ± SEM	*p* values	mean difference ± SEM	*p* values
**IL-6**	390 ±134	< 0.05	593 ± 254	< 0.05
**MCP-1**	317 ± 101.1	< 0.05	612.2 ± 107	< 0.01
**FN1**	225 ± 47.2	< 0.05	337 ± 67.3	< 0.01
**Type I collagen**	11.07 ± 2.04	< 0.01	17.4 ± 1.67	< 0.001
